# Supplementary material for: Discovery of Sustainable Energy Materials Via the Machine‐Learned Material Space
Source: Small. 2025 May 5;22(12):2412519. doi: 10.1002/smll.202412519 (PMC12934376; doi:10.1002/smll.202412519)
Supplement: Supplementary file 1 — Supporting Information [file SMLL-22-2412519-s001.pdf]

Supporting Information for "Discovery of Sustainable Energy Materials via the Machine-Learned Material Space"

Malte Grunert,[1,2,a)] Max Großmann,[1,2] and Erich Runge[1]

[1)] *Institute of Physics and Institute of Micro- and Nanotechnologies, Technische Universität Ilmenau, 98693 Ilmenau, Germany*

[2)] *These authors contributed equally to this work.*

(Dated: 3 April 2025)

As supporting information, we provide a list of the numerical values of sustainability and criticality used in the main work. In addition, we show versions of Fig. 1 with additional colorations as well as a discussion of them, UMAPS resulting from a different network architecture, and equivalent t-SNE visualizations for Figs. 1-4 of the main text. Interactive versions of the UMAP plots shown in this paper and a Jupyter notebook for creating custom UMAP plots will be provided to reviewers as supplemental material and will be made publicly available upon publication.

NUMERICAL VALUES OF SUSTAINABILITY AND CRITICALITY

Figure S1 shows the sustainability and criticality values used in the main text, as extracted from the European Chemical Society[1] and the European Commision's Joint Research Centre,[2] respectively. Regarding the criticality values, all elements not listed in Ref. 2 are assumed to have a criticality of zero. In addition, some elements are not directly named in their elemental form - we map carbon to 'natural graphite', silicon to 'silicon metal', fluorine to 'fluorspar', and potassium to 'potash'.

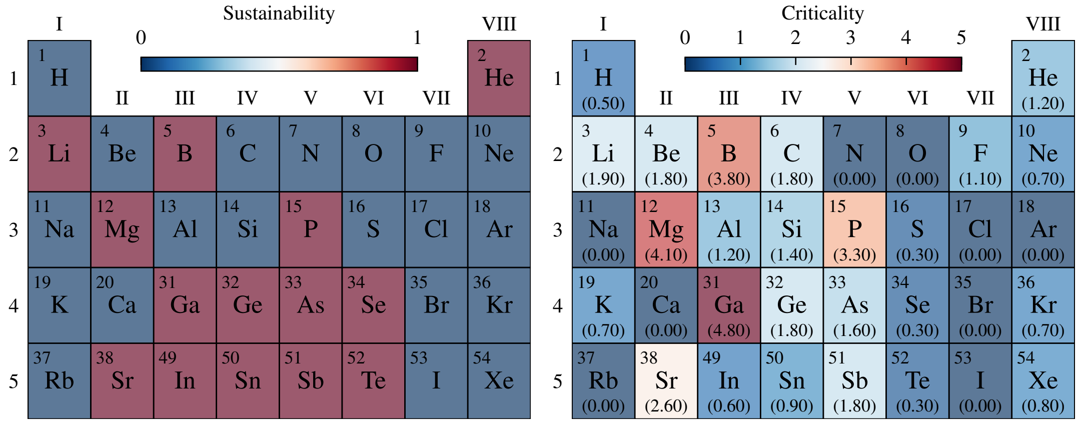

FIG. S1. Left: Sustainable elements as in Ref. 1. Elements that are considered as 'sustainable' are blue, while 'unsustainable' elements are red. Right: Criticality of elements as in Ref. 2, Table 13. Higher values indicate a larger supply chain risk, i.e., 'criticality'.

ADDITIONAL UMAPS WITH DIFFERENT COLORATIONS

As Fig. S2, we show a version of Fig. 1 of the main text, colored according to the occurrence of different elements, namely sulfur (S), selenium (Se), tellurium (Te), and iodine (I). Even without a more detailed examination, some interesting observations can be made: For example, in the right upper area are some clusters containing sulfur, selenium, and tellurium in a reasonable order (S to Se to Te), but no oxygen, although it is in the same group (see Fig. 1 of the main text). It is a well-known chemical fact that oxygen behaves quite differently from the other group VI elements. Curiously, in the central upper region there is a cluster with significant overlap between sulfur and selenium,

a) Electronic mail: malte.grunert@tu-ilmenau.de

with tellurium covering a separate region of the same cluster. Upon closer investigation using the interactive UMAP, the present authors, who are not specialists for group-VI chemistry, found no easily discernible reason why this should be the case. Also interesting, and the reason we included iodine in this figure, is the cluster on the left. A comparison with Fig. 1 in the main text shows that this is the cluster of oxoanions, and indeed we find compounds containing, e.g., SO_3, SO_4, or SeO_4 ions there. However, there is also a region where compounds containing SeO_6 (orthoselenate, to our knowledge only reported once in the literature) and TeO_6 (orthotellurate, well known) are present, as well as, e.g., compounds with IO_6 (orthoperiodate, well known). We believe that it is rather unlikely that such similarities and differences are even known to most chemists, let alone physicists.

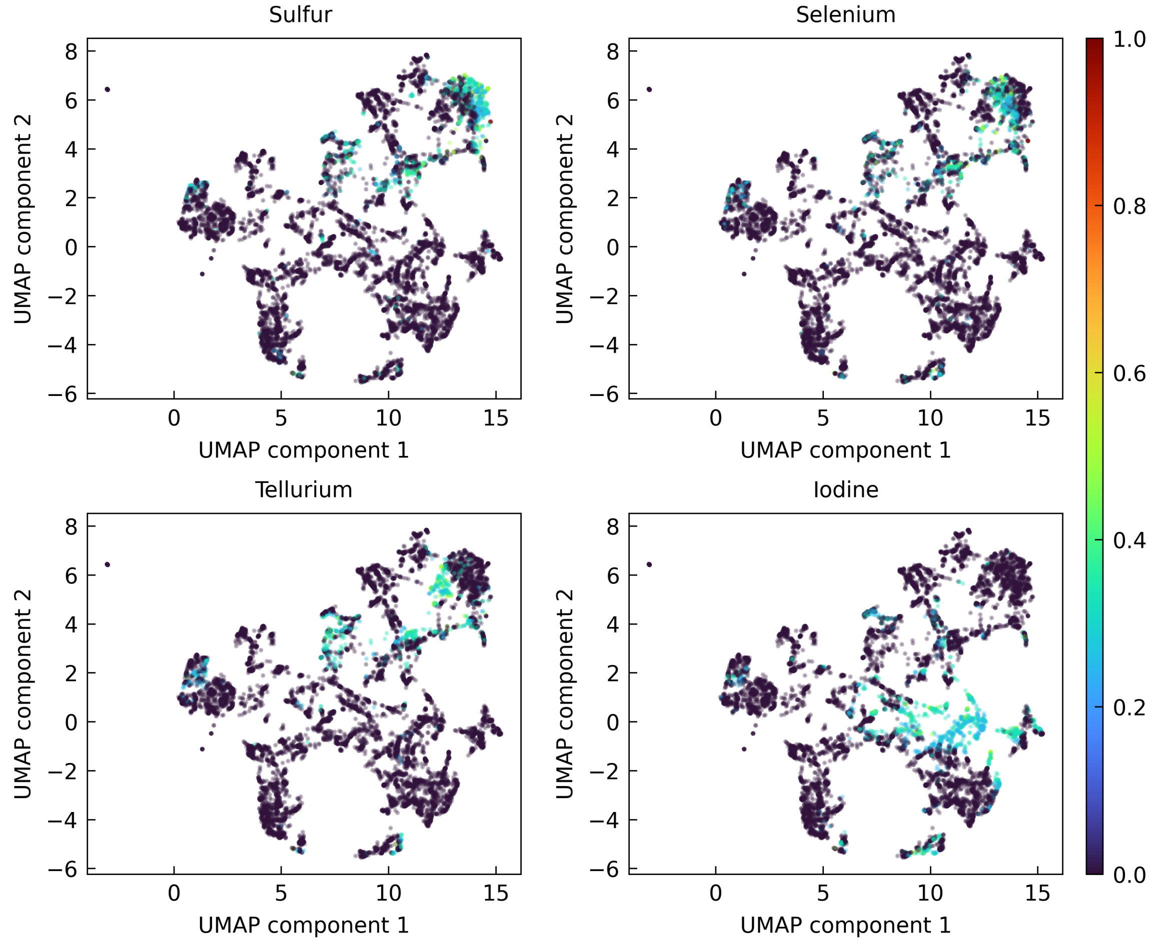

FIG. S2. Extension of Fig. 1 from the main text. UMAPs of the latent embeddings after the pooling operation, colored according to the prevalence of certain elements: (top left) sulfur, (top right) selenium, (bottom left) tellurium, (bottom right) iodine. The colorbar indicates the percentage of all atoms in each material that belong to the given element.

In Fig. S3, we show a coloration according to nitrogen and carbon contents. Both elements are positioned close to each other and relatively close to the large oxoanion cluster (see Fig. 1 of the main text). The overlap between both elements consists of compounds containing both carbon and nitrogen, often in the form of cyanide or cyanamide ions, while the overlap between the oxoanion cluster and carbon consists of compounds containing e.g., carbonate, and the overlap between the oxoanion cluster and nitrogen consists of compounds containing e.g., nitrate and nitrite. In the center of the general carbon, nitrogen, and oxygen regions, alkali cyanates (e.g., NaCNO) are found. This again highlights the remarkable chemical intuition present in OptiMate.

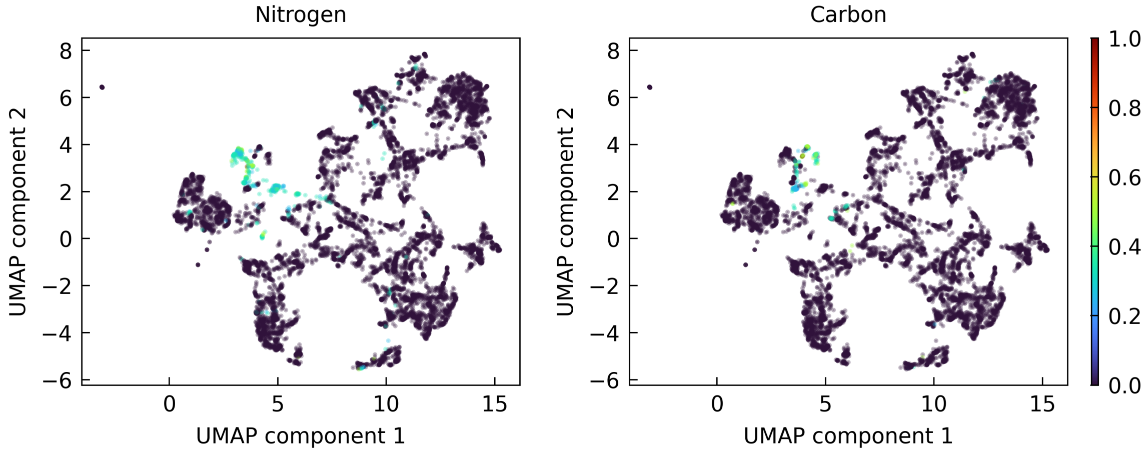

FIG. S3. Extension of Fig. 1 from the main text. UMAPs of the latent embeddings after the pooling operation, colored according to the prevalence of certain elements: (left) nitrogen, (right) carbon. The colorbar indicates the percentage of all sites in each material that belong to the given element.

UMAPS FOR DIFFERENT NET ARCHITECTURES

To address the question, how universal the characteristics of the presented UMAPs are, we compare two different models trained on the same data. The left panel of Fig. S4 reproduces for convenience data from Fig. 1 and Fig. S3, whereas the right panel shows results for the same GAT architecture (Fig. 1a) but with different hyperparameters, in particular changed embedding dimensions. For easier comparison, we applied a so-called procrustes transform (centering, rotating and linearly scaling the data points so that the maps are orientated similarly).
As the UMAP algorithm is non-linear, the resulting UMAP can visually look significantly different, even for models with similar latent representations.[3] Nonetheless, if two models learn the same physical features, and therefore represent the same materials as either similar or different, the resulting UMAPs should preserve this as well. In other words: While the global orientation and scale of the UMAP plot are essentially meaningless and change from run to run,[3] the contents of each cluster, and according to which property clusters are formed/not formed should be consistent. The location of clusters with respect to each other, especially far-away clusters, can however also be expected to change from model to model. Indeed, one can see that while the global arrangement and the shape of the clusters changes, clustering according to the presence of elements remains the same. The models, regardless of the specific hyperparameters, appear to learn, e.g., that oxygen and nitrogen are very important elements for the optical properties, while halogenides or alkali earths do not play such an important role. Furthermore, even some of the global topology is reserved: For example, the nitrogen-containing clusters are close to the oxygen-containing clusters in both UMAPs.

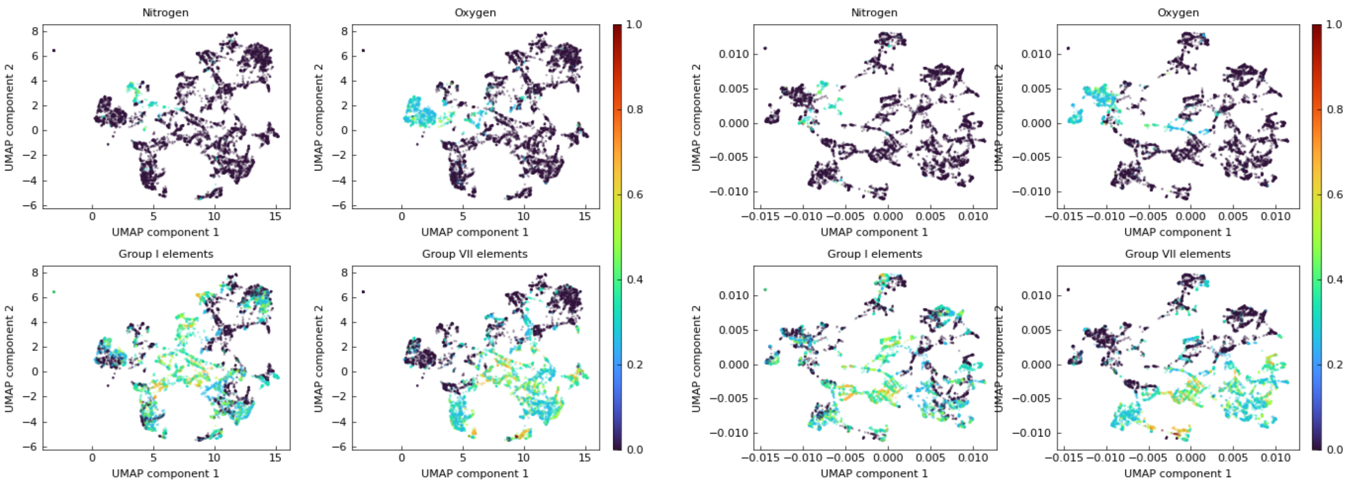

FIG. S4. Comparison between UMAPs resulting from different architecture hyperparameters. Left: Original model, as shown in the paper. Right: Model with significantly reduced number of parameters, after procrustes transform. While the overall position of clusters differs, the elements according to which clusters are formed remain the same across different model architectures.

T-SNE VISUALIZATIONS

In Figs. S5-S8, we show t-SNE visualizations of the embeddings corresponding to the UMAPs in Figs. 1-4 of the main paper. t-SNE is another dimension reduction algorithm, similar to UMAP. The t-SNE plots were generated using the default settings in the Python package *scikit-learn*. The differences between t-SNE and UMAP are summarized well in Ref. 3. The general clustering behavior, especially those of different elements, is similar to that observed in the UMAP plots, further supporting the notion that the observed clustering is a result of truly meaningful internal representations. The comparison of our t-SNE and UMAP results is not inconsistent with the general expectation that UMAP better preserves global distances,[4] but since the original space is very high dimensional, this is difficult to confirm directly.

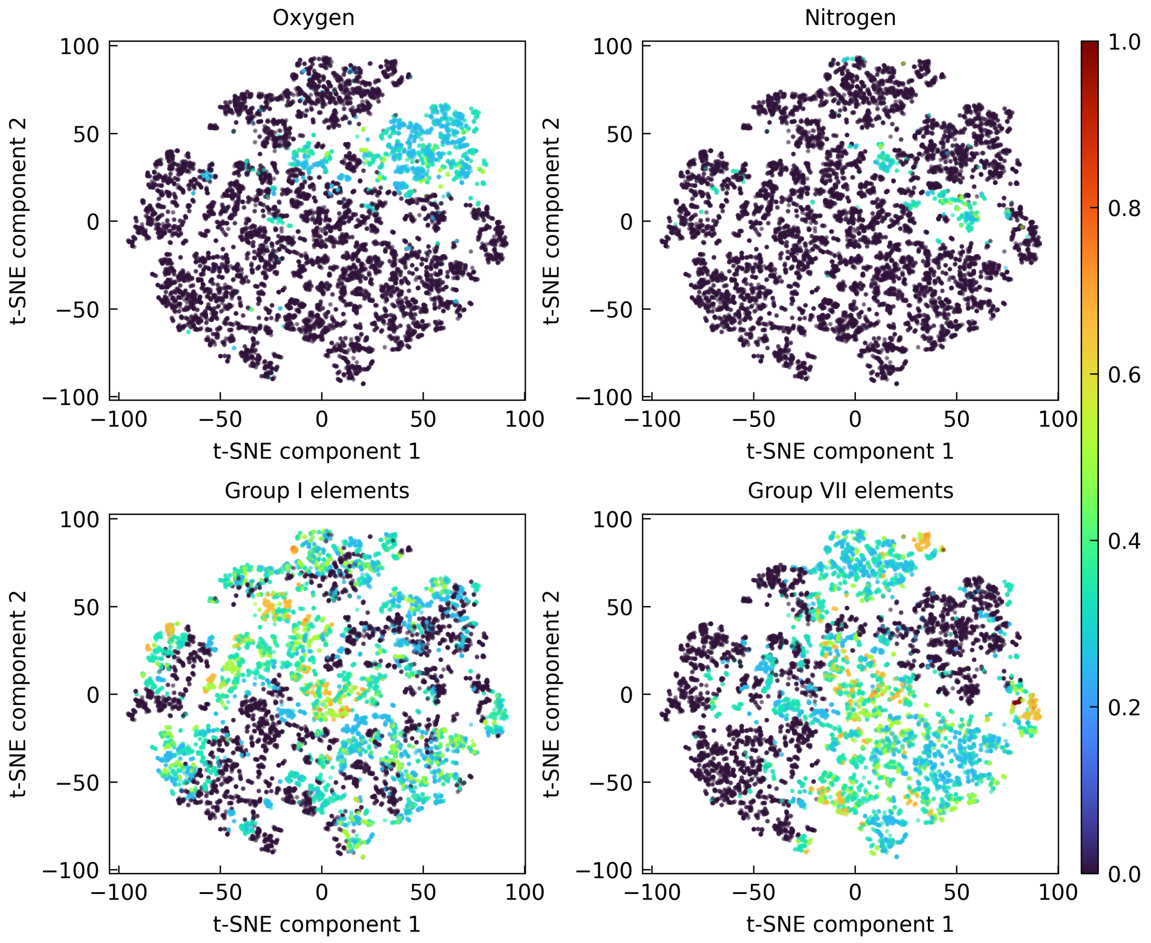

FIG. S5. t-SNE of the latent embeddings after the pooling operation, colored according to the prevalence of certain elements: (top left) oxygen, (top right) nitrogen, (c) group I elements, (d) group VII elements.

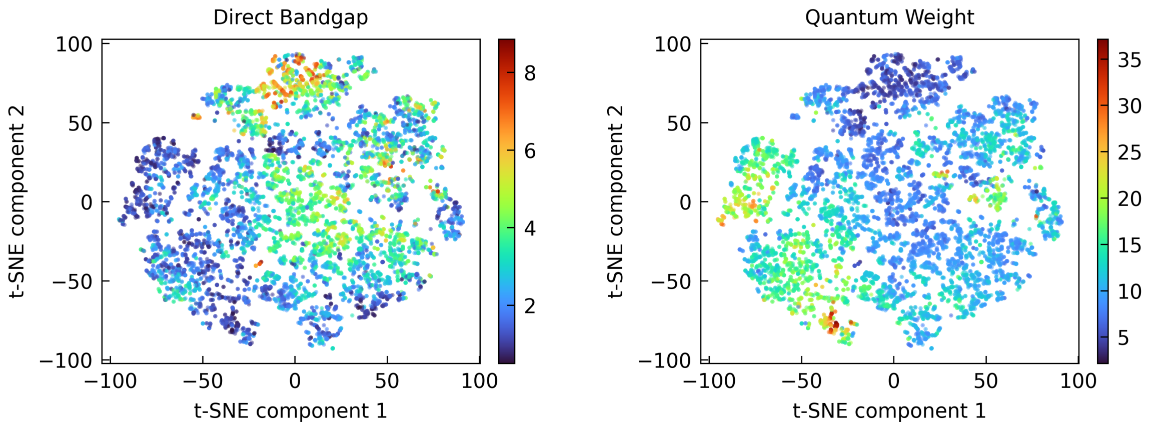

FIG. S6. t-SNE of the latent embeddings after the pooling operation, colored according to (left) the direct band gap as obtained from DFT and (right) the quantum weight calculated from the predicted spectra.

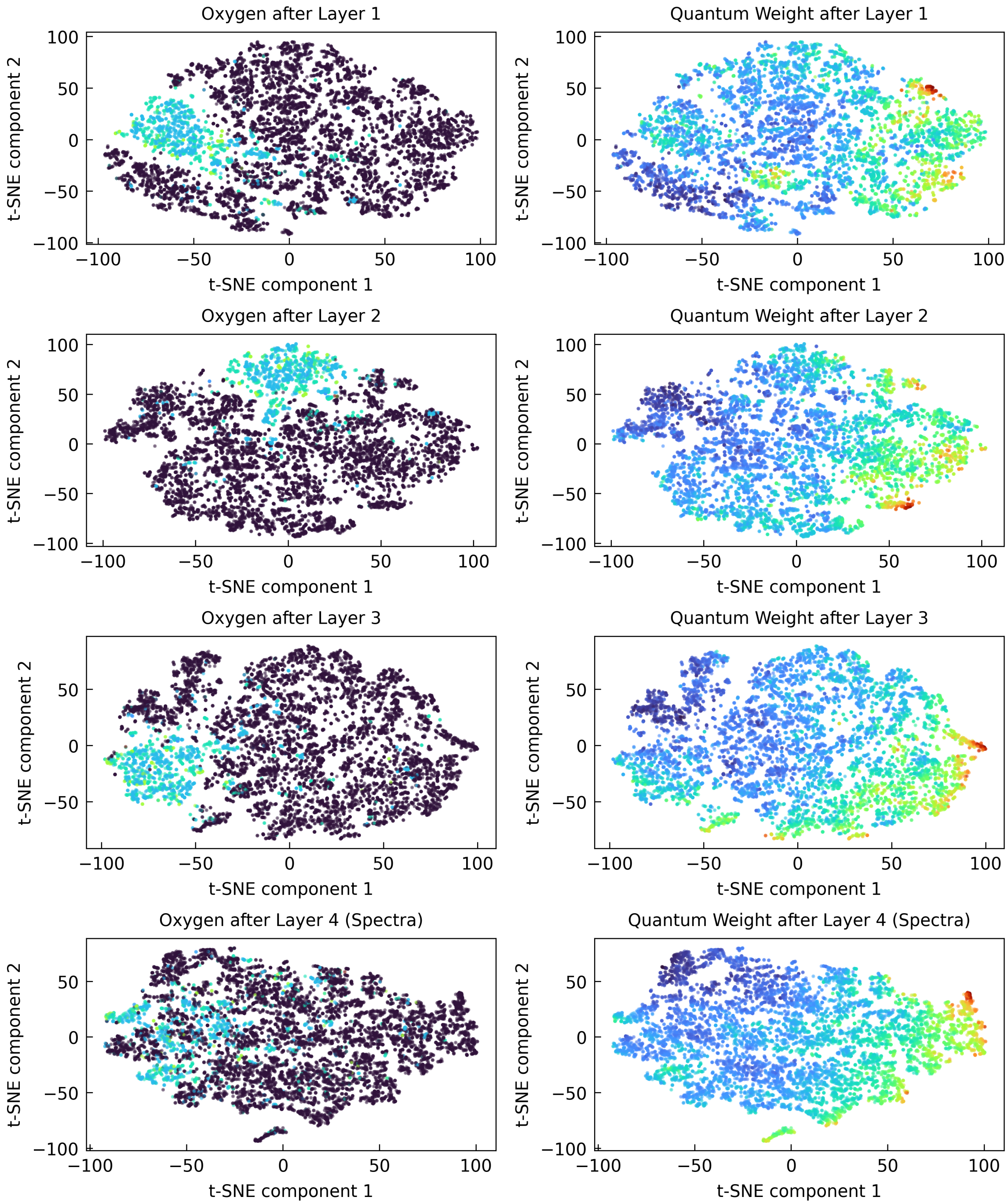

FIG. S7. t-SNE of the latent embeddings after the first (top), second (middle), and third (bottom) layer of the specta prediction MLP, colored according to (left) the share of oxygen in the compounds and (right) the quantum weight calculated from the predicted spectra.

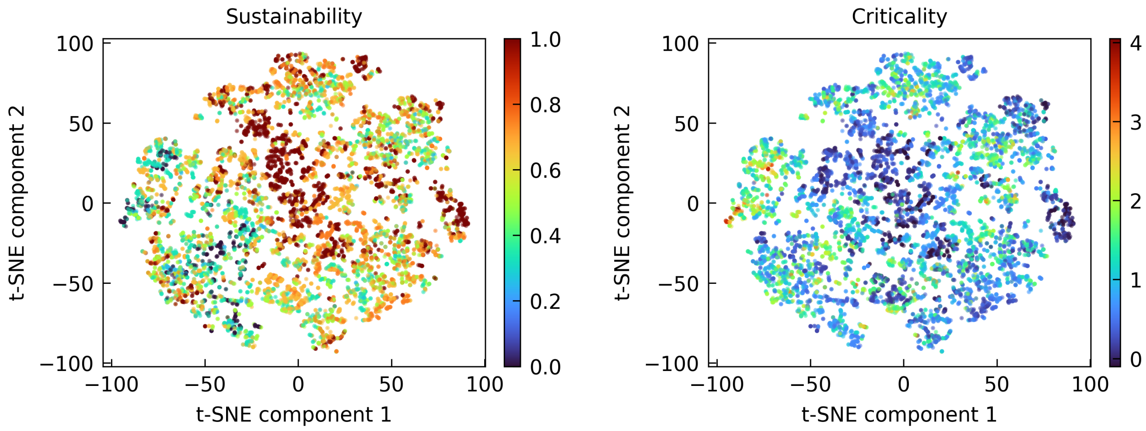

FIG. S8. t-SNE of the latent embeddings after the pooling operation, colored according to (left) the sustainability and (right) the criticality. Higher values correspond to less sustainable/more critical materials.

[1] European Chemical Society, "Element Scarcity - EuChemS Periodic Table - EuChemS — euchems.eu," `https://www.euchems.eu/euchems-periodic-table/` (2024), [Accessed 20-11-2024].

[2] European Commission, Joint Research Centre, S. Carrara, S. Bobba, D. Blagoeva, P. Alves Dias, A. Cavalli, K. Georgitzikis, M. Grohol, A. Itul, T. Kuzov, C. Latunussa, L. Lyons, G. Malano, T. Maury, A. Prior Arce, J. Somers, T. Telsnig, C. Veeh, D. Wittmer, C. Black, D. Pennington, and M. Christou, *Supply chain analysis and material demand forecast in strategic technologies and sectors in the EU – A foresight study* (Publications Office of the European Union, 2023).

[3] L. McInnes, J. Healy, and J. Melville, "UMAP: Uniform Manifold Approximation and Projection for Dimension Reduction," (2018), arXiv:1802.03426.

[4] A. Coenen, A. Pearce, and Google PAIR, "Understanding UMAP — pair-code.github.io," `https://pair-code.github.io/understanding-umap/` (2024), [Accessed 21-11-2024].
